# Insights Into Mucosal Innate Immune Responses in House Dust Mite-Mediated Allergic Asthma

**DOI:** 10.3389/fimmu.2020.534501

**Published:** 2020-12-07

**Authors:** Arwa Abu Khweek, Eunsoo Kim, Marisa R. Joldrichsen, Amal O. Amer, Prosper N. Boyaka

**Affiliations:** ^1^Department of Biology and Biochemistry, Birzeit University, Birzeit, Palestine; ^2^Department of Microbial Infection and Immunity, The Ohio State University, Columbus, OH, United States; ^3^Department of Veterinary Biosciences, The Ohio State University, Columbus, OH, United States; ^4^The Infectious Diseases Institute, The Ohio State University, Columbus, OH, United States

**Keywords:** mucosal, innate immunity, asthma, house dust mite, allergy

## Abstract

The prevalence of asthma has been rising steadily for several decades, and continues to be a major public health and global economic burden due to both direct and indirect costs. Asthma is defined as chronic heterogeneous inflammatory diseases characterized by airway obstruction, mucus production and bronchospasm. Different endotypes of asthma are being recognized based on the distinct pathophysiology, genetic predisposition, age, prognosis, and response to remedies. Mucosal innate response to environmental triggers such as pollen, cigarette smoke, fragrances, viral infection, and house dust mite (HDM) are now recognized to play an important role in allergic asthma. HDM are the most pervasive allergens that co-habitat with us, as they are ubiquitous in-house dusts, mattress and bedsheets, and feed on a diet of exfoliated human skin flakes. *Dermatophagoides pteronyssinus*, is one among several HDM identified up to date. During the last decade, extensive studies have been fundamental in elucidating the interactions between HDM allergens, the host immune systems and airways. Moreover, the paradigm in the field of HDM-mediated allergy has been shifted away from being solely a Th2-geared to a complex response orchestrated via extensive crosstalk between the epithelium, professional antigen presenting cells (APCs) and components of the adaptive immunity. In fact, HDM have several lessons to teach us about their allergenicity, the complex interactions that stimulate innate immunity in initiating and perpetuating the lung inflammation. Herein, we review main allergens of *Dermatophagoides pteronyssinus* and their interactions with immunological sentinels that promote allergic sensitization and activation of innate immunity, which is critical for the development of the Th2 biased adaptive immunity to HDM allergens and development of allergic asthma.

## Introduction

Asthma is a chronic lung disease characterized by airway obstruction, mucus production and bronchospasm that affects approximately 339 million people worldwide (1). It is a cause of both premature death and reduced quality of life in individuals of all ages in all parts of the world. Not only the prevalence of asthma is increasing, but it continues to be a major public health and global economic burden due to both direct and indirect costs (2). The definition of asthma has evolved over the last three decades as our understanding of the pathophysiology and different clinical presentation has developed. During the twentieth century, researchers recognized constriction of airway smooth muscles and excessive sensitivity of the airway to external stimuli (hyper-responsiveness) as the key features of asthma. In the 1980s, it was established that airway inflammation was an essential feature, with structural changes in the airways (remodeling) present early in the development of the disease. Currently, it is well-accepted that asthma is a heterogeneous inflammatory disease associated with airways narrowing, swelling and excessive mucus production. It is also defined by the history of respiratory symptoms such as intermittent attacks of breathlessness, bronchoconstriction, coughing, wheezing, dyspnea, and airway inflammation (3–5).

## House Dust Mite Allergens and Their Roles in Asthma

House dust mites are small yet, extremely complex organisms that belong to the group of arthropods and thrive in humid indoor environment. They come in contact with humans mainly through mattresses and bedsheets, and live on a diet of skin scales and bio-debris accumulated in house dust. Hypersensitivity to HDM such as *Dermatophagoides pteronyssinus* aeroallergens contribute to atopic sensitization in 50–85% of asthmatics, and are strong inducers of allergenicity worldwide (6–8). In addition to genetic predisposition, mite sensitization, and exposure are important determinants of the subsequent development of asthma (3–5). Thus, patients with asthma and dust mite sensitivity show worsened bronchospasm and bronchial hyper-reactivity following exposure to mite allergen, while reduced symptoms are noted in a mite-free environment (3). The link between HDM and asthma is further supported by the fact that the levels of HDM exposure and sensitization are strong predictors for asthma in clinics (9, 10). Furthermore, the synergy between mite sensitization, exposure and respiratory viral infection enhances the severity of the disease, and is the leading cause of acute wheezing or hospitalization (11–13). Other pieces of evidence that link HDM and asthma include the fact that inhalation of HDM allergens induces the proliferation of bronchial smooth muscle (14), and that HDM-specific IgE were detected in the sputum of asthmatics (15).

The high percentage of HDM-mediated allergy encouraged extensive research on HDM allergens. An array of at least 30 allergen have now been identified for *D. pteronyssinus* (16, 17). They are classified according to a Linnaean system for nomenclature based upon their genus, species, and the order they were discovered (18). For example, Der p 1 was the first discovered allergen from the genus *Dermatophagoides*, species *pteronyssinus* (19). Originally, HDM allergens were classified into major and minor groups based on their allergenicity and strict IgE reactivity. The major group allergen Der p 1 and Der p 2, and minor group allergen Der p 4, Der p 5 and Der p 7 account for ~50 and 30% of HDM-specific IgE, respectively (17). Thus, these major groups are serodominant (19, 20), while other groups have less clinical relevance (21–23). More recently, Der p 23, a peritrophin-like protein, has been identified as another major HDM allergen of *D. pteronyssinus* (24–27). Although Der p 23 is only present in low amounts in house dust and in HDM extracts, it was shown to induce IgE-dependent basophil activation which indicated a high allergenic activity (28). A recent study analyzed the profile of sensitization to HDM major components. The authors found that most individuals in the pediatric population with respiratory allergy disease with mite allergy are sensitized and contained IgE specific to Der p 1, Der p 2, and Der p 23 major allergens (29). The clinical relevance Der p 23 was further established by the fact that 5% of this population was mono-sensitized to Der p 23 and presented clinical symptoms of HDM allergy (29).

Nowadays, the complexity of HDM components and their mechanism of action led to an updated classification that matches their biological activity and regulatory effects on the epithelial cells, and cells of the innate and adaptive immune systems. *D. pteronyssinus* have allergens that exhibit proteolytic activity, homology with the lipopolysaccharide-binding component of Toll-like receptor 4, homology with other invertebrate tropomyosins, and chitin-cleaving and chitin-binding activity (30, 31). The major allergens of *D. pteronyssinus* are listed in Table 1.

**Table 1:** HDM allergens derived from *Dermatophagoides pteronyssinus*.

**Allergens**	**Biological functions**
Der p 1	Cysteine protease
Der p 2	Lipid binding
Der p 3	Serine protease (trypsin)
Der p 4	Amylase
Der p 5	Lipid binding
Der p 6	Serine protease (chymotrypsin)
Der p 7	Lipid binding
Der p 8	Glutathione transferase
Der p 9	Serine protease (collagenase)
Der p 10	Muscle tropomyosin
Der p 11	High molecular weight muscle paramyosin
Der p 12	Homology to chitin
Der p 13	Lipid transfer
Der p 14	Lipid transport
Der p 15	Chitinase
Der p 16	Gelsolin
Der p 17	EF hand protein
Der p 18	chitin-binding protein
Der p 20	Arginine kinase
Der p 21	Putative fatty acid binding
Der p 23	Peritrophin, chitin binding

## Airway Epithelium: the Initiator and Regulator of HDM-Mediated Allergic Asthma

**Figure 1:**
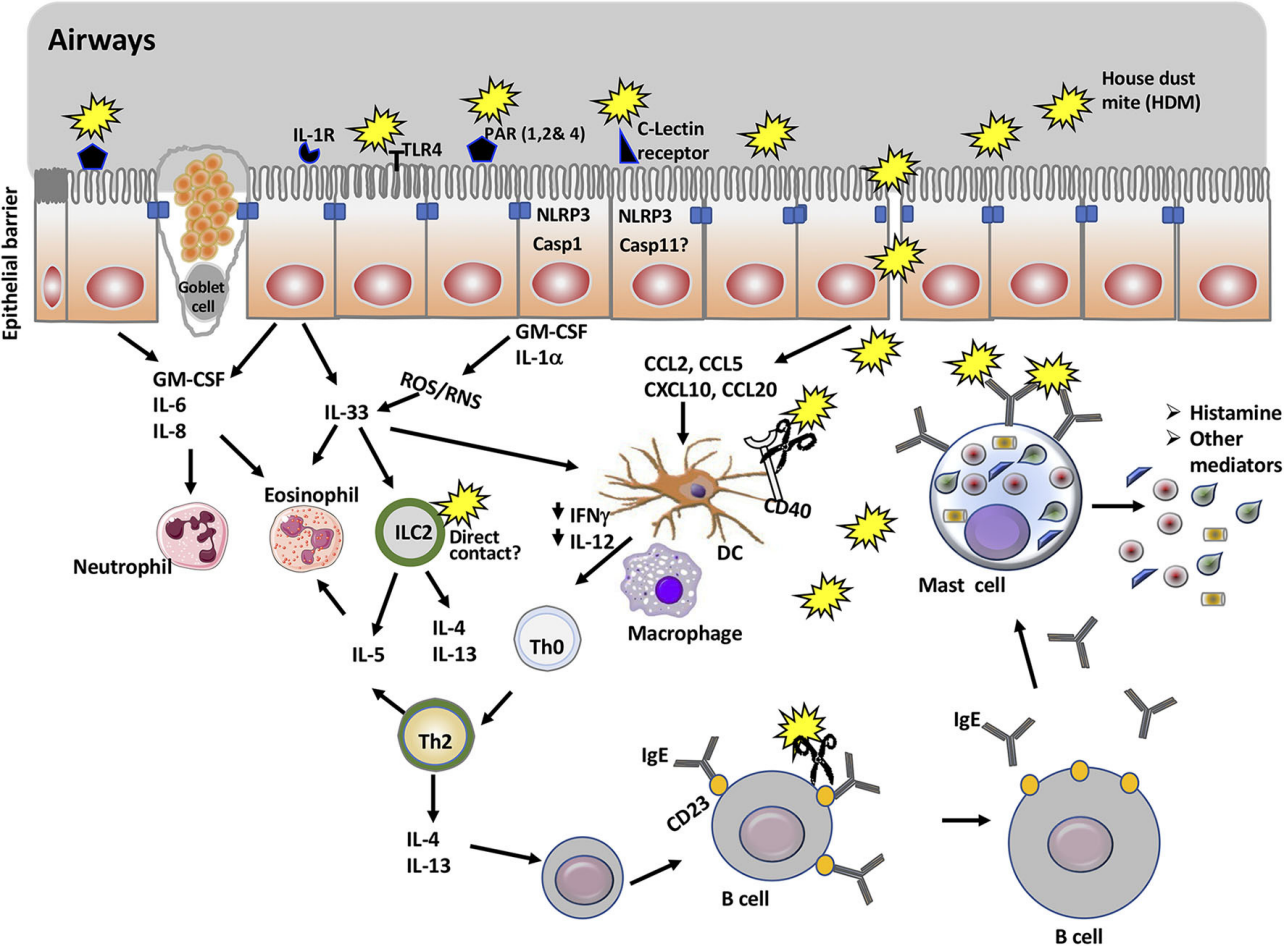
Overview of mucosal immune cells, molecules, and pathways targeted by house dust mite proteins to promote allergic asthma. The main allergen of house dust mite (HDM) is Der p 1, a cysteine protease. In addition to being an antigen, Der p 1 promotes allergic asthma through its action on a number of immune cells and molecules. Thus, Der p 1 can cleave tight junction proteins of epithelial cells and gain access to immune cells including naïve dendritic cells. It can also activate protease sensitive receptors (PARs) and TLR4, or promote cell injury in epithelial cells. This will result in the activation of NLRP3 inflammasome and secretion of cytokines and chemokines that recruit myeloid cells including dendritic cells, eosinophils, and ILC2. Cleavage of CD40 limits the production of IL-12 and IFNγ and thus, favors the polarization of Th2 cells. Der p 1 enhances the production of IgE though cleavage of CD23 receptor, which disrupts the negative feedback for regulation of IgE production. Finally, cross-linking of Der p 1-specific IgE on mast cells by Der p 1 mediates mast cell degranulation and release of histamine and other mediators associated with the symptoms of the allergic asthma.

Initial interactions with immunological sentinels start when HDM allergens reach and interact with the airway epithelial cells and immune cells resident in the airway lumen (Figure 1). Increasing evidence demonstrates that asthma is an epithelial cells disorder and that its clinical manifestations are associated with compromised epithelial integrity (32, 33). Epithelial cells are joined by tight junction protein complexes which act as a fence to avert the passage of exogenous molecules and microbes from the airway lumen into the blood. The proteolytic activity of HDM allergens, which is limited to a distinct part of the allergome, is what makes them powerful. Although HDM harbor a repertoire of allergens with protease activity, those of group 1 or initiator (e.g., Der p 1) are the chief responsible for activating the innate immune response and facilitating polysensitization to allergens from unrelated sources (34).

Inhibition of Der p 1 proteolytic activity abolished allergic sensitization in mice challenged intranasally with HDM (35). In fact, the protease activity of Der p 1 targets tight junction proteins such as Occludin and Claudins and thus, increases the barrier permeability of the airway mucosa (36, 37) and subsequent influx of allergens (19, 35, 36, 38). Other groups of HDM allergens are triad of serine proteases (Der p 3, Der p 6, and Der p 9) that exhibit sequence homology to trypsin, chymotrypsin, and collagenase, respectively (39, 40). Serine proteases cleave tight junction proteins and exhibit redundancy with group1 in their functionality, thus their role in allergic sensitization is subordinate (35).

Winton et al. used five cell lines to study the effects of proteinases on epithelial permeability (41). Intriguingly, HDM proteinases of both cysteine and serine classes were able to perturb epithelial cells adhesion and function (35, 40, 41). However, the cell cultures were performed in the absence of serum and cells could lack essential growth factors required for expression and function of intercellular junctions and the cytoskeleton. Thus, a challenge in working with epithelial cell lines *in vitro* appears to be insuring the provision of an environment in which airway epithelial cells will optimally express the desired characteristics, and a normal repertoire of functional intercellular adhesion molecules. In fact, significant discrepancy can occur between crude measurements of “epithelial leakiness” (uncorrected solute clearance) and more precise measures of permeability under conditions where changes in physiological function occur. Furthermore, experiments performed in serum free media do not account for factors that can limit the activity of HDM proteases, *in vivo*.

Zhang et al. used specific inhibitors of proteases including serine proteases and cysteine proteases, to elucidate the enzymatic activities of Der p 1 that played a major role in allergic sensitization (42). These studies have shown that the serine proteases of Der p1, or the closely related Der f 1 (a major allergen of *D. farinae*), only play a minor role in allergic sensitization. On the other hand, the cysteine peptidase activity of Der p 1 is central to the initiation of allergy because its bioactivity mediated both the degradation of tight junctions and the polarization of immune responses toward Th2-type. Thus, the cysteine peptidase activity Der p 1 favors both allergen delivery and IgE production (42). However, the unrestrained contribution from HDM serine peptidase allergens is insufficient to drive these responses when Der p 1 is inhibited. Thus, endogenous serine peptidase inhibitors which are known to be vulnerable to degradation by Der p 1 (43, 44) provide protection against them when the activity of Der p 1 is inhibited. In fact, Der p 1 is an enabler/initiator of allergy to unrelated proteins as it provides a means to cross the epithelial layer and overcome mucosal tolerance.

Previous studies have shown that Der p 1 facilitates sensitization to ovalbumin and that the potentiation of allergenicity is dependent on the protease activity of Der p 1 (45, 46). Although it is capable of initiating IgE synthesis, ovalbumin differs markedly from Der p 1 as the former requires an adjuvant to overcome tolerance when delivered to the airways. Therefore, the protease activity of Group 1 HDM must act similarly for unrelated allergens of mite origin. The adjuvant effect of Group 1 HDM allergens on the overall sensitization to HDM allergens is only one example of their ability to promote allergic responses to bystander allergens of non-HDM origin. Therefore, it is possible that the serine proteases alone lack the adjuvant activity needed to overcome the immunological tolerance toward antigens delivered into the airways.

Even though HDM proteases cleave tight junction proteins, desmosomal adhesion are examples of junctions that are not a primary cleavage target of HDM proteases (36). Cleavage of tight junction proteins by HDM proteases is one mode by which they compromise epithelial permeability. Other indirect effects of HDM allergens include decreased expression of tight junction proteins (33, 47).

Beside physical defenses provided by epithelial cells, the airway surface liquid is rich in antiprotease molecules such as α1-antitrypsin, which display a new facet of interactions with protease allergens. Thus, in general, allergen serine proteases are inhibited by α1-antitrypsin. However, when the mite feces reach the lower airways, Der p 1 inhibit the anti-protease-based lung defense by cleaving the α1-antitrypsin (43, 44). Interestingly, Der p 1 by itself is resistant to most antiproteases (43, 44). Furthermore, Der p 1 degrades pulmonary surfactants (SP-A and SP-D) in the airway surface liquid that would normally bind inhaled allergens and prevent them from reaching IgE bound to mast cells (48, 49).

Since HDM are ubiquitous in the environment and can be found as contaminants in food, several groups investigated the effect of HDM on the gastrointestinal (GI) tract. Thus, ingestion of contaminated food can lead to intestinal inflammation (50). Tulic et al. detected Der p 1 in the intestinal fluid of asthmatic patients and its concentration gradually decreased along the GI tract with higher concentration in the duodenum and the lowest in the colon (51). HDM was also reported to impair intestinal barrier function and increase intestinal permeability by reducing the expression of tight junction proteins such as zonula occludens protein 1 (ZO-1) and occludin-1, as well as the production of mucus (51). Cysteine protease activity of HDM induced pro- and anti-inflammatory cytokines, and it was aggravated in the inflammatory bowel disease (IBD) patients (51). It remains unclear how ingestion of HDM could impact allergic sensitization to HDM or allergic responses upon exposure to HDM via the airways.

## Innate Responses Induced by HDM Allergens

In addition to cleaving tight junctions and compromising epithelial cell permeability, dust mite proteases orchestrate a series of signaling events within the sphere of innate immunity (Figure 1). Innate immunity is a highly sophisticated system of defense mediated by immune cells in responses to signals they receive through a variety of pattern recognition receptors (PRRs). Whether they are extracellular or intracellular, PRRs sense pathogen-associated molecular patterns (PAMPs) (e.g., LPS, chitin) and danger-associated molecular patterns (DAMPs) (e.g., uric acid, ATP) that are released during tissue injury (52). Extended epithelial PRRs activation by allergens or via microbial PAMPs contaminants represent one of the central steps in Th2-mediated sensitization which results in the release of proinflammatory cytokines and chemokines, thus amplifying the influx of Th2 cells, DCs, eosinophils, basophils, and other inflammatory cells (53, 54). Further, released cytokines activate innate immune cells such as basophils, mast cells, eosinophils and group 2 innate lymphoid cells (ILC2) to sustain the Th2-mediated inflammation (55). Stimulated ILC2 release Th2 cytokines such as IL-13 and IL-5 (55). Therefore, Th2-mediated response is a result of complex crosstalk between the epithelium and cells of the innate and adaptive immunity.

### Stimulation of Protease-Activated Receptors (PARs)

In addition to increasing epithelial cell permeability, dust mite proteases stimulate protease-activated receptors (PARs) in the bronchial epithelium of asthmatics (56). Unexpectedly, Der p 1 has been shown to exhibit prothrombinase activity, thus converting prothrombin to thrombin (57). Canonical activation of PAR-1 and PAR-4 is mediated via HDM serine proteases such as Der p 3, or Der p 9. However, non-canonical activation of these receptors by Der p1 through its prothrombinase activity promoted release of reactive oxygen (ROS), nitrogen species (RNS), respectively (42, 57). Even though serine proteases are the candidates for activation of PARs, selective inhibition of Der p 1 attenuated production of reactive species. This suggests that Der p 1 is the main allergen stimulating the release of these reactive species. Downstream activation of PAR-1 and PAR-4 by Der p 1-thromin axis result in pannexons opening leading to release of ATP (57, 58). ATP is considered a DAMP that activates A disintegrin of metalloproteases 10 (ADAM 10), thus mediating the production of ROS (58). In the airway epitheilal cells, ADAM 10 initates further conversion of prothrombin to thrombin downstream of the prothrombinase activity of Der p 1, thus positively feedback ROS production (59, 60). Consistently, ATP and thrombin levels are elevated in asthma (61, 62). The fact that Der p 1 mediated innate ROS production through a thrombin dependent mechanism sheds new light on how allergens directly influence the development of Th2-biased immune responses (57, 58). Importantly, other studies have reported an imbalanced reducing and oxidizing (redox) environment favoring oxidation is present in asthma (63). Such an oxidative environment could modify proteins and alter their biological functions needed for initiation and maintenance of inflammation. This can include the loss of antioxidant capacity of the superoxide dismutase (SOD) that catalyzes the reaction of superoxide to hydrogen peroxide and the transformation of hydrogen peroxide into water (63).

Cunningham et al. examined the importance of the cysteine protease activity by activating papain, a plant cysteine proteases, that is homologous to Der p 1. Papain was activated through reduction with L-cysteine, and compared with the non-reduced papain for their ability to induce allergic inflammation (64). Challenge with the activated papain markedly increased IgE and IgG1 antibody responses as well as in eosinophilia and IL-4, IL-5, and IL-10 in the BAL of mice. Further, the enzymatic activity of low doses of cysteine protease allergens administered to the respiratory mucosa drive Th2 immune responses that can mediate fulminant lung hypersensitivity responses and persist in the face of ongoing exposure. Therefore, it is essential for Der p 1 to be activated by reduction in order to overcome the oxidative environment in the lungs and mediate allergic sensitization. Generated reactive species induce histone modifications and the activation of redox-sensitive transcription factors which promote pro-allergic cytokines. They further activate signaling pathways such as mitogen-activated protein kinases (MAPK), and the signal transducer and activation of transcription (STAT) family that is associated with allergy and asthma (65, 66). In addition, epithelilal ROS production modulates the release of IL-33, a pivotal cytokine that acts on ILC2 to promote a Th2-biased response by dendritic cells (67). Although humans are continuously exposed to HDM allergens, only a subset of the population become sensitized. People at higher risk of sensitization to HDM allegens exhibit genetic defect in the antioxidant defenses or in the susceptibility to ROS/RNS which are triggered by the innate cellular responses to these allergens (63, 68, 69). Indeed, the antioxidant enzymes superoxide dismutases (SOD) and catalase are reduced in asthma as compared to healthy individuals, with lowest levels in those patients with the most severe asthma (63).

Further, HDM allergen-mediated PARs activation promotes the release of pro-Th2 cytokines and chemokines including IL-6, IL-8, and GM-CSF in cultured airway epithelial cells. These cytokines and chemokines trigger the extravasation and accumulation of eosinophils, basophils, and neutrophils, which perpetuate the allergic inflammation of the airways (21, 70–76). Moreover, activation of PARs by peptide agonist that mimics PARs ligands induce the release of cytokines suggesting that, at least the serine proteases promote the allergic response via induction of cytokine release (71, 76). One of the PAR family receptor, PAR-2 is upregulated by airway epithelium, and its expression correlated with asthma severity and airway smooth muscle enlargement (77–79). Several lines of evidence indicate that activation of PAR-2 plays a key role during the sensitization phase of the HDM allergy model, leading to increased lung cytokine production and augmented lung reactivity (80). First, PAR-2 deficient mice sensitized and challenged with HDM fail to develop airway inflammation and HDM-specific IgG1. Second, blocking PAR-2 activation diminishes Th2-mediated cytokines, airway hyperresponsiveness, and airway inflammation in response to HDM challenge (80). Third, PAR-2 activation contributes to the development of IgE response, thus promoting adaptive immunity to HDM (81). However, studies demonstrated that release of cytokines by epithelial cells in response to Der p 1 is PAR-2 independent (70, 74). Even though, PAR-2 is involved in the response to HDM allergens; it was shown that PAR-2 is dispensable for allergy development by inhaled route in PAR-2 deficient mice (81). In addition, PAR-2 inhibit eosinophilia, airway hyperresponsiveness and bronchoconstriction in a murine model of allergic inflammation (82), or enhances airway inflammation and airway hyperresponsiveness (72). Therefore, PAR-2 can exert cytoprotective or inflammatory effects based on the circumstances.

### Activation of TLR4

Toll-like receptor 4 (TLR4) is one member of the TLR family that is expressed on airway epithelial cells and has drawn attention in the context of allergy due to its ability to bind LPS. LPS from Gram-negative bacteria is the most studied PAMP and considered as contaminant of HDM allergens. The importance of TLR4 in HDM-mediated allergy was shown through epidemiological and mechanistic studies. Epidemiological studies demonstrated that high LPS dose is detrimental in allergic sensitization and development of asthma, which is inversely proportional with the level of LPS contained in the HDM (83, 84). Low doses of LPS promote Th2 response and eosinophilic inflammation while high doses mediate Th1 response and neutrophilic inflammation (85, 86). Elegant mechanistic studies revealed that the expression and triggering of TLR4 on epithelial cells are indispensable for sensitization to HDM allergens (87–89). The link between TLR4 activation by contaminating LPS and HDM sensitization was established by showing that LPS promoted IL-1α release (88). Secreted IL-1α alarmin targets epithelial cells in an autocrine manner, to release GM-CSF and IL-33 which recruits dendritic cells (87, 88), and neutralization of these triad cytokines dampens allergy development (88). Importantly, release of IL-33 is ROS-regulated by NADPH oxidase dual oxidase 1 (DUOX1) whose activity is elevated in allergic disease (90). Further, ablation of TLR4 on epithelial cells or inhibition through antagonists reduced recruitment and activation of sub-epithelial DCs and suppressed features of asthma such as bronchial hyperreactivity (87, 88). In addition, knockout mice in *TLR4* and *MyD88* are attenuated in Th2 cytokines, IL-17, neutrophilia, and bronchial hyperreactivity compared to WT (91–94). These data indicated that HDM, specifically LPS regulate Th2 and Th17 associated with lung inflammation. Even though inhibition of the activated cysteine protease inhibited (but did not abolish) most of sensitization, responses could be still dependent on TLR4 especially under chronic exposure of low dose allergens.

Several studies have shown a crucial role of TLR4 in sensitization to aero-allergens (85, 87) and especially in sensitization to Der p 2 (20) via the broncho epithelial route. The central role of TLR4 in HDM sensitization was shown to be dependent upon group 2 allergens such as Der p 2, which exhibit similarity to myeloid differentiation antigen-2 (MD-2), a TLR4 co-receptor (20). It was thought that MD-2 mimicry could compensate for the lack of MD-2 expression in the epithelial cells, thus establishing a mechanistic link to the role of LPS in allergy. Indeed, MD-2 expression in cultured primary epithelial cells is associated with increased responsiveness to LPS (95). Trompette et al. used a model of sustained hypersensitivity to assess the role of TLR4 and MD-2 receptor in allergic sensitization. The authors used intranasal injections for 21 days to address whether the induction of asthma by Der p 2 is TLR4- and MD-2 dependent (20). Initial assessment of the involvement of TLR4 and MD-2 in Der p 2 mediated signaling was performed *in vitro* using HEK293 cells. Trompette et al. showed that the mutation of tyrosine to alanine (Y91A) ablated the ability of Der p 2 to reconstitute LPS-driven IL-8 production in HEK293 cells expressing TLR4 without MD-2, or to augment LPS-driven IL-8 production in HEK293 cells co-expressing TLR4 and MD-2. Thus, in HEK293 cells, Der p 2 promotes LPS-induced TLR4 signaling in a manner that appears to depend on Der p 2 and TLR4 interactions. Sensitization and challenge with Der p 2, along with extremely low (pg range) of LPS, induced robust airway Th2 inflammation-marked by airway eosinophilia and lymphocytosis, mucous metaplasia, and increased plasma IgE concentrations in wild type but not TLR4-deficient mice. Notably, Der p 2 similarly induced Th2 inflammation in the airways of MD-2-deficient mice. On the other hand, mutant Der p 2(Y91A) failed to induce experimental allergic asthma. Thus, *in vivo* allergenic activity mirrors *in vitro* functional and biochemical activity: Der p 2 efficiently drives airway Th2 inflammation *in vivo* in a TLR4-dependent manner, and retains the ability to drive such inflammation in the absence of MD-2. Mutations in LPS that would abrogate the binding to TLR4 would reduce the allergic sensitization.

In contrast to data generated for the lung, Stremnitzer et al. showed that TLR4 does not account for sensitization to Der p 2 in the skin (96). Indeed, TLR4^−/−^ mice showed an even stronger overall Th2-biased antibody response to epicutaneous Der p 2 than WT mice receiving the same treatment. In contrast to lung models (20, 87), sensitization to Der p 2 was even more pronounced in TLR4^−/−^ mice if LPS was present. Therefore, Der p 2 alone can cause a strong Th2-biased response via the skin, which is further enhanced in the presence of LPS, in TLR4-independent manner. Furthermore, TLR4 expression does not enhance but rather protect against epicutaneous sensitization to house dust mite allergen Der p 2. These findings suggest that the skin is an important sensitization route that may differentially modulate immune responses to allergens than after exposure via the bronchial epithelium. In this regard, functional TLR4 might have a protective function against skin allergens. It is also possible that the response unaffected by the cysteine protease would become important with low dose chronic exposure experienced by humans.

Importantly, group 2 allergens exhibit hydrophobic pocket capable of binding LPS and potentially bind to lipids other than LPS and activate other TLRs. However, the indispensability for TLR4 in HDM sensitization and allergy does not seem to be merely dependent on MD-2 mimicry for several reasons. First, group 2 allergens do not induce ROS production and have no direct effect on the epithelium unless they are administered in conjunction with group 1 HDM allergens. Second, selective inhibition of Der p 1 proteolytic activity abolishes signs of allergic sensitization in mice, an effect that is hard to associate with the importance of TLR4 being absolutely dependent on MD-2 mimicry (35). More insight into the role of TLR4 in HDM-mediated allergy was obtained by showing that TLR4 activation by HDM mediates ROS production is Der p 1-dependent (42). Der p 1 mediates TLR4 activation by putative endogenous ligands such as fibrinogen (97), and selective inhibition of Der p1 abolished ROS production (34). The Der p 1-dependent activation of TLR4 is an outcome of signaling events initiated by its activation of PAR-1 and PAR-4. The authors show that TLR4 ligation to ROS production occurs downstream from the gating of pannexons and endogenous activation of prothrombin (42). Therefore, stimulation of PRRs by HDM plays a pivotal role in allergic sensitization and development of asthma. Even though Der p 1 and Der p 2 have been studied in the context of innate immune stimulation through TLRs, putative interactions of other HDM allergens with PPRs need to be elucidated.

### Activation of NOD-Like Receptors (NLRs)

The cytosolic NOD-like receptors (NLRs) protein family such as NLRP3 or NLRC4 sense PAMPs or DAMPs. Pattern sensing is followed by nucleation and oligomerization of the adaptor protein ASC (apoptosis-associated speck like protein containing CARD), the engagement of the cysteine protease pro-caspase-1, and formation of the multiprotein inflammasome complex (98–100). Canonically within the oligomerized inflammasome complex, dimers of pro-caspase-1 undergo proteolytic auto-cleavage into the enzymatically active caspase-1, which consequently catalyzes the final processing of the inflammatory cytokines pro-IL-1β and pro-IL-18 precursors into their mature and secreted forms (101). To date, contradictory results were obtained regarding the role of NLRP3 in HDM-mediated allergy and the role of other NLRs in asthma is poorly understood. Early studies showed that NLRP3 is dispensable for HDM-induced allergic airway inflammation (102). Intranasal challenge with HDM extract promoted uric acid release, a prototypic activator of the NLRP3 inflammasome (103). Intriguingly, uric acid is elevated in asthma patients and has been shown to promote Th2 allergic inflammation in response to HDM (102). The adjuvant effect of uric acid is independent of NLRP3-ASC-IL-1-MyD88 axis, but associated with activating dendritic cells via spleen tyrosine kinase and PI3-kinase δ signaling (102). However, recent study showed that the NLRP3 complex modulates HDM-induced inflammation by controlling eosinophil influx, and Th2 cytokines and chemokines in the airways of mice. Therefore, *NLRP3*^−/−^ mice exhibit increased airway inflammation in response to HDM, which is associated with augmented immune cell infiltration specifically eosinophils; Th2 cytokines and chemokines in their airways (104). This study further demonstrated that mice lacking *caspase-1* exhibit robust lung inflammation characterized by eosinophilia and an increase in IL-33, TSLP, and IL-25 cytokines (104). Augmented lung inflammation is attributed to increased expression of IL-33, uric acid and spleen tyrosine kinase production (105). In addition to lacking caspase-1, mice used in this study are also deficient in caspase-11, another cysteine protease that is related to caspase-1 and activated non-canonically (106). Therefore, it is not known whether the observed phenotypes are attributed to the lack of caspase-1 or caspase-11 or both.

Caspase-11 or (caspase-4/5 in humans) belongs to the family of inflammatory caspases that share homology with caspase-1 (107–114). Caspase-11 participates in the activation of the NLRP3 inflammasome and mediates pyroptotic cells death that is associated with the release of inflammatory IL-1β and IL-18 in response to non-canonical stimuli (106, 115–117). While caspase-1 is constitutively expressed, caspase-11 expression is induced through LPS-activated TLR4 signaling via the adaptor TIR-domain-containing adaptor-inducing interferon-β (TRIF) and TRIF-dependent type I interferon production (99, 106, 118). However, the roles of caspase-11 in HDM-mediated asthma need to be elucidated.

HDM-mediated allergy has a significant reliance on bioactive constituents acting in synergy to produce their biological effects. However, commercial extracts can vary in their composition which affects their bioactivity profiles. Therefore, the conflicting results regarding the role of NLRP3 in HDM-mediated allergy could arise from using different HDM extracts, dosage, route and length of HDM challenge. In the ovalbumin model, absence of NLRP3 was associated with reduced allergic inflammation in the presence or absence of aluminum hydroxide (alum) adjuvant (119, 120). The difference seen between HDM and ovalbumin could account for the difference in antigenicity which is associated with different cytokines that are involved in aggravating the airway inflammation (104).

Two independent studies have shown that caspase-11-deficient mice are resistant to developing experimental allergic airway inflammation in response to two different allergens. Using HDM, we showed a global reduction in inflammation in the lungs of *caspase-11*^−/−^. This reduction is manifested by reduced cellular infiltration including neutrophils, macrophages and lymphocytes in the lungs. We also observed reduced Th1, Th2, and Th17 cytokines in the broncho-alveolar lavage fluid derived from *caspase-11*^−/−^ mice (Abu Khweek et al., under review). The reduced lung inflammation in *caspase-11*^−/−^ could be due to increased level of IgA, which potentially neutralizes HDM in our experimental model. It can also be due to reduced migration of neutrophils to the lungs in response to lower levels of KC and IL-17A. Alternatively, it may be due to reduced IL-33 that can lead to reduced IL-4 and IL-5 production by T cells. Our study offers several intriguing scenarios for the diverse functions of caspase-11. Expression of caspase-11 in innate immune cells following HDM exposure may be required for the antigen presentation by macrophages and dendritic cells to naïve T cells. Subsequently, insufficient antigen presentation leads to reduction in cytokines and chemokines released in the BAL fluids. Our published work and that of others also support the notion of inherent defect in migration of *caspase-11*^−/−^ cells due to impaired actin cytoskeleton (121, 122). Zaslona et al. showed that *caspase-11*^−/−^ mice injected and challenged with ova plus alum are resistant to developing allergic airway inflammation. The reduced inflammation is associated with lessened infiltration of leukocytes to the lungs, especially eosinophils. In addition, *caspase-11*^−/−^ mice showed reduced release of Th2, Th1, Th17 cytokines, and circulating IgE (123). Even though we used a different allergen, exposure time and routes of administration, the findings by Zaslona are in line with several of our data. Taken together, these studies demonstrate that caspase-11 activity is important for the induction of allergic asthma.

### Interactions With ILCs

Group 2 innate lymphoid cells are a recently discovered innate immune cells that are closely related to allergen responses as these cells produce the same cytokines as Th2 cells (124). They are the most predominant ILC population in the lungs at steady state, and when activated they expand rapidly (125). Also, according to experiments performed by Smith et al., the number of ILC2s is higher in the peripheral blood mononuclear cells (PBMC) of allergic asthma patients when compared to healthy donors, and even donors with allergies but no asthma (126). In general, ILC2s are activated by IL-33, IL-25, and thymic stromal lymphopoietin (TSLP) which induce them to rapidly expansion and produce IL-4, IL-5, and IL-13 (124). This is however contrasted by the way that ILC2s seem to react in cases of HDM exposure. Thus, it was suggested that T cells are necessary for complete ILC2 function in an HDM model of allergic asthma in mice (127). While underlying mechanisms are not fully understood, this report further illustrates the complexity of the HDM allergic asthma model, and the interconnectedness of the innate and adaptive immunity in this complex disease scenario.

### Regulation of Myeloid and Epithelial Cells

Beyond the disruption of major junctional adhesions of epithelial cells, the proteases released by HDM facilitate allergens access to submucosal tissues and their detection by APCs such as dendritic cells (DCs) (128). Recruitment of APCs at mucosal surfaces and their departure for interaction with naive T cells is instrumental in bridging innate and adaptive immune signaling to allergens. It is now appreciated that the role of DCs is beyond sensitization to HDM allergens. In fact, DCs are fundamental in instructing humoral and Th2-mediated responses and their increased functionality could be proportional to epithelium permeability (128). Moreover, differentiation of naïve T cell into Th1, Th2, or Th17 is dependent upon DCs- derived cytokines (128). Der p 1 promotes migration of DCs into the bronchial epithelial in response to production of chemokine C-C ligand 2 (CCL2), CCL5, CCL20, and CXCL10 by bronchial epithelial cells (71, 75, 129). The release of CCL20 is Der p 1- and TLR4-dependent (73, 87). In addition, stimulation of the epithelial airways by HDM allergens and release of CCL20 could be induced via a non-Toll pathogen recognition receptor such as C-lectin (130). The induction of CCL20 secretion is specific for HDM, and no other aeroallergens, and is independent of TLR4/2 and proteases (130). Instead, secretion of CCL20 is mediated via ligation of β-glucan moiety present in the HDM to the C- type lectin receptor dectin1 (130). Further demonstration that β-glucan is the ligand inducing CCL20 by epithelial cells was shown through abrogation of HDM mediated release of CCL20 by β-glucanase or competitive inhibition with β-glucans. The relevance of C-lectin receptor to HDM-mediated allergy is reinforced by evidence that dectin-1 is critical for the development of HDM-induced influx of eosinophils, neutrophils into the lungs, and the generation of Th2 cytokines (131, 132). Dectin-1 is specifically expressed on CD11b^+^ DCs, an essential subset of DCs in allergic sensitization, but not on other DCs or lung epithelial cells. Dendritic cells elicit a superior capacity for antigen uptake and presentation that is attributed to expression of the C-lectin receptor dectin-1. Therefore, activation of this specific subset of DCs by HDM β-glucan in the lung could couple innate and adaptive immunity (132). Moreover, stimulation of dectin-1 or mannose receptors by chitin, a repeating units of β-(1–4)-poly-N-acetyl-D-glucosamine mediates Th1, Th2, and Th17 polarization as well as the recruitment of eosinophils and basophils (133, 134). However, the molecular mechanisms by which chitin promote innate immunity need to be elucidated.

An large body of literature has focused on the direct injury caused by the protease activity of allergens, including damaging tight junctions and subsequent activation of the signaling pathways (56, 135, 136). However, downstream of airway epithelial injury and initiation of signaling events, HDM allergens must directly interact with APCs, a key event to promote adaptive immunity. Allergens interactions with DCs are mediated via mannose receptor and DC-SIGN which belong to the C-lectin receptors. These receptors mediate uptake of glucan structure of Der p 1, Der p 2 resulting in Th2 polarization (137, 138). In addition, Der p 1 downregulates the expression of tryptophan catabolizing enzyme, indoleamine 2,3-dioxygenease (IDO) in DCs (137). Immunomodulatory functions of IDO include the induction of regulatory T cells (Tregs) or the depletion of effector T cells, which have been shown to inhibit the development of asthma in mouse models (139, 140). Furthermore, downregulation of IDO by Der p 1 could inhibit immune tolerance and lead to development of allergic sensitization. Therefore, therapeutics designed to manipulate IDO activity could promote tolerance and inhibit allergic sensitization.

## Adaptive Immunity to HDM

The high allergenicity of HDM proteases is also associated with their ability to mediate Th2 biased immune response by reducing Th1 polarization (141, 142). Der p 1-mediated Th2 cell differentiation through IL-6 production by epithelial cells, and limiting innate signals required for Th1 differentiation (143). Der p 1 promotes Th2 biased proliferation by deconstructing the IL-2 receptor which is involved in mediating Th1 proliferation (142). In addition, compelling evidence indicate that the proteolytic activity of Der p 1 is instructional in directing DCs-mediated Th2 subset development. Therefore, Der p 1-promoted DCs activation induced Th2 polarization via reduced release of IL-12, a pivotal cytokine in Th1 polarization and differentiation (144). Der p 1-induced suppression of IL-12 production is due to cleavage of CD40, a co-stimulatory molecule expressed on APCs, thus rendering DCs less responsive to CD40-CD40L pathway (144). Furthermore, DCs that mature in presence of proteolytically active Der p 1 and reduce the release INF-γ, but increase production of IL-4 by CD4^+^ T cells, thus promoting Th2 polarization (144). The resulting cytokine milieu again favors the polarization of naïve T cells into Th2 cells.

Effector functions of B cells include production of HDM-specific immunoglobulins, of which IgG, IgA, IgM, and IgE are released in response to allergens. Although IgE exerts a pathogenic function, allergen specific-IgA or IgG4 are thought to have protective functions by capturing and neutralizing allergens at mucosal surfaces (145–147). Further dimension to the pleotropic role of Der p1 was demonstrated via disrupting the negative feedback loop for IgE-production in B cells which eventually results in the progression of the allergic disease (48, 148). Der p 1 activates the signaling cascade associated with ADAM10, a “sheddase” of the low affinity IgE receptor, cluster of differentiation 23 (CD23) (48, 148, 149). Proteolytic release of CD23 from cells is considered a key event in allergic asthma. Soluble CD23 promotes antigen presentation to allergen-specific B cells, thus mediating the formation of allergen-specific IgE. Excessive production of IgE inhibits the negative feedback mechanism that would otherwise restrict IgE production (48, 148).

Besides immunoglobulin production, B cells exhibit accessory roles in antigen presentation especially when T cells are initially primed by DCs following allergens capturing through the surface immunoglobulin receptor (150, 151). Even though B cells are not as efficient in antigen presentation, their presence in T cell-inductive sites and production of cytokines that influence the activation of DCs and naïve T cells and their expression of costimulatory molecules such as CD80 and CD40 ligand equip B cells with antigen presenting and other accessory functions that are pivotal for promoting and sustaining Th2 immunity. *In vitro*, activated B cells from HDM-exposed mice presented antigen to T cell receptor transgenic mice (1-DER) T cells and induced a Th2 phenotype. *In vivo*, B cell contribution to the induction of Th2 inflammation and asthmatic features have been shown to be dependent on HDM allergen dose and on the tissue site at which Th2 cytokine is probed. Only under low dose of allergens, B cells become necessary for optimal expansion of HDM-reactive Th2, central memory T (T_CM_) cells, and for development of eosinophilic airway inflammation and bronchial hyperreactivity (152). Therefore, HDM proteases target different substrates that are required in various immune mechanisms starting from epithelial barrier function, proceeding to innate inflammatory responses, involving T cell polarization, and even have a direct effect on IgE production by B cells.

## Therapeutic Approaches to HDM-Induced Allergy

HDM avoidance is the first recommended method to reduce the symptoms in clinic now. However, in Cochrane meta-analyses on mite avoidance, the use of environmental control measures was found to be of little benefit in reducing rhinitis symptoms, and with no effect on alleviating asthma symptoms (153, 154). In addition to allergen avoidance, pharmacotherapy is also part of the treatment. These therapeutic approaches primarily aimed at controlling inflammation of the upper and lower airways employ antihistamines, leukotriene receptor antagonist, and/or inhaled/intranasal corticosteroids (155). Although these phamacological treatments are effective and safe in most cases, they rarely change the course of HDM-related allergic diseases.

Allergen immunotherapy (AIT) consists of administration of small doses of allergen. This therapeutic approach has been used for the past century as subcutaneous immunotherapy (SCIT), and more recently as sublingual immunotherapy (SLIT). In contrast with pharmacotherapy, effective AIT activates multiple mechanisms. Firstly, SCIT reduces allergen specific IgE production and increases the production of specific IgG (which acts as a “blocking” antibody) (156). Secondly, AIT induces a major change in allergen-specific T-cell subsets, including immunologic deviation (stimulation of Th0/Th1 lymphocytes, with increased IFN-γ and IL-2 production), specific T-lymphocyte anergy (a decrease in Th2/Th0 lymphocyte counts), and induction of regulatory T-lymphocytes, which produce cytokines such as IL-10 and TGF-β. Thirdly, suppression of peripheral ILCs, especially ILC2s, might contribute to Th2 suppression and immunologic tolerance (157). Lastly, AIT decreases inflammatory cell recruitment, activation, and the release of mediators including histamine, prostaglandin D2, and eosinophil cationic protein (158). All these effects of AIT contribute to immune tolerance and long-lasting changes in the immune system, even after the treatment is discontinued. The mechanisms of SLIT are not fully understood but they seem to be similar to those of SCIT, except that in SLIT, mucosal dendritic cells are particularly involved in this process (159). A prolonged duration of treatment is required for long-term efficacy after discontinuation of immunotherapy (160). AIT was previously shown to prevent the development of new sensitizations in HDM monosensitized children (161). The European Academy of Allergy and Clinical Immunology (EAACI) guidelines on AIT for allergic asthma and the new Global Initiative for Asthma (GINA) guidelines now include HDM SCIT (162). It is also worth noting that recent SLIT-based AIT trials in house dust mite (HDM) allergic asthma have shown some efficacy. Thus, sublingual HDM tablet AIT were found to reduce asthma exacerbations and enabled steroid reduction. Accordingly, the new GINA 2020 guidelines recommend SLIT therapy for HDM-related allergic asthma for individuals with mild asthma (FEV1 > 70%) (163). The selection of allergen components, total number of allergens, and relative proportions of individual allergen components included in therapeutic mixtures are critical aspects of formulating allergen immunotherapy. When preparing mixtures of allergen extracts, the prescribing physician must take into account the cross-reactivity of allergen extracts and the potential for allergen degradation caused by proteolytic enzymes (164). AIT has been defined by a WHO leading paperas as “the only form of treatment able to modify the natural course of allergic diseases” (165), and it is the only potential disease-modifying treatment of HDM allergic subjects. Both SCIT and SLIT with HDM vaccine show safety and efficacy in reducing symptoms and medication usage, and in improving quality of life for treatment.

The clinical significance of group 1 HDM allergens and their serodominance as allergens as well as their activities in priming the innate immune system make them as an attractive target for therapeutic intervention. Resolving the structure of allergens and their earlier molecular interactions with immunological sentinels will pave the way for designing novel therapy used in the future prevention and treatment of asthma.

## Conclusions And Perspectives

The interactions between allergens, immune responses, and airway diseases are highly complex and still incompletely understood. Studies during the last decade were instrumental in casting the light on the role of HDM in allergy and atopy, in general. More specifically, they educated us about the allergenicity of HDM, its structural components, its proteases and its interaction with the innate and the adaptive immune systems. However, HDM extracts used in experimental studies are man-made and often intra-nasally administered in large volumes of solution. Thus, they may vary from HDM in the environment by their allergen composition and the presence of other environmental substances. In this regard, the allergenicity of dust mites is not solely dependent on the structural components of independent mite proteins such as Der p 1 since biological fluids and proteins in general block its proteolytic activity and therefore, its allergic sensitization potential. In fact, group 1 allergens from HDM are digestive enzymes excreted in fecal pellets which are inhalable by humans. The accumulation of these microscopic fecal pellets on the airway surface achieves a high localized concentration of the enzyme and reduces their resistance to proteolytic inactivation (37).

New advancements resulted in a paradigm shift in the field of asthma and it is increasingly appreciated that in addition to IgE, asthma is mediated by complex responses orchestrated via extensive crosstalk between the epithelium, professional APCs and cells of the adaptive immunity. These interactions are mediated via environmental allergen exposure and can be influenced by several factors including genetic predisposition, polymorphism, and the status of epithelial cell integrity or viral exposure. Dissecting the role of HDM allergens has helped in understanding the role of innate immunity and shed the light on its relevance in initiating and perpetuating the lung inflammation. Given the large number of HDM allergens and apart from group 1, 2, 3, and 9, our knowledge is still lacking regarding mechanisms utilized by allergens to trigger the immune system. Characterization of the molecular basis of allergenicity and the functional bioactivities of these allergens will pave the way for identification of novel therapeutics (37, 166). Furthermore, successful drug design would selectively target various aspects of HDM-epithelial cell interactions, which could potentially inhibit the driving signal for activating the innate system that is required to sustain adaptive immunity. Drugs that inhibit the release of epithelial cell derived cytokines would inhibit HDM-induced allergic sensitization.

## Author Contributions

AA, AOA, and PB: planned the manuscript. AA, EK, MJ, and PB: wrote the manuscript. AOA and PB: edited the manuscript. All authors contributed to the article and approved the submitted version.

## Conflict of Interest

The authors declare that the research was conducted in the absence of any commercial or financial relationships that could be construed as a potential conflict of interest.
